# An Unusual Case of a Young Woman with a Postnasal Space Mass

**DOI:** 10.4269/ajtmh.17-1013

**Published:** 2018-07

**Authors:** Shuwei Zheng, Kok Hing Lim

**Affiliations:** 1 Department of Infectious Disease, Singapore General Hospital, Singapore, Singapore;; 2 Department of Anatomical Pathology, Singapore General Hospital, Singapore, Singapore

**Figure 1.**
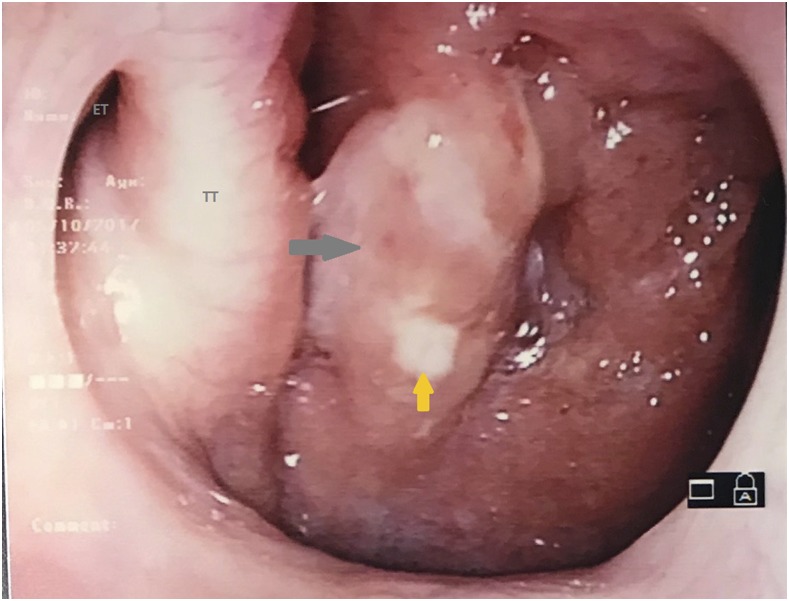
Postnasal space mass seen on nasopharyngoscopy (gray arrow shows a mass in the right postnasal space; orange yellow shows mucopurulent material; ET = eustachian tube; TT = torus tubarius). This figure appears in color at www.ajtmh.org.

A 23-year-old woman, without any background medical history, presented to the otolaryngology clinic with voice hoarseness after an upper respiratory tract infection. Her clinical history began 3 months ago with fever and cough, followed by persistence of voice hoarseness and postnasal drip symptoms. She had been an avid singer but had stopped because of the change in her voice. There was no history of weight loss or appetite loss. She was a smoker of one pack a year and had not reported any significant social, exposure, or travel history. Physical examination was unremarkable without any cervical lymphadenopathy or pulmonary findings. Laboratory evaluation revealed normal full blood count, and renal and liver function. Nasopharyngoscopy demonstrated the presence of a mass in the postnasal space associated with mucopurulent discharge (Figure 1). The rest of the upper airway was unremarkable.

**Figure 2.**
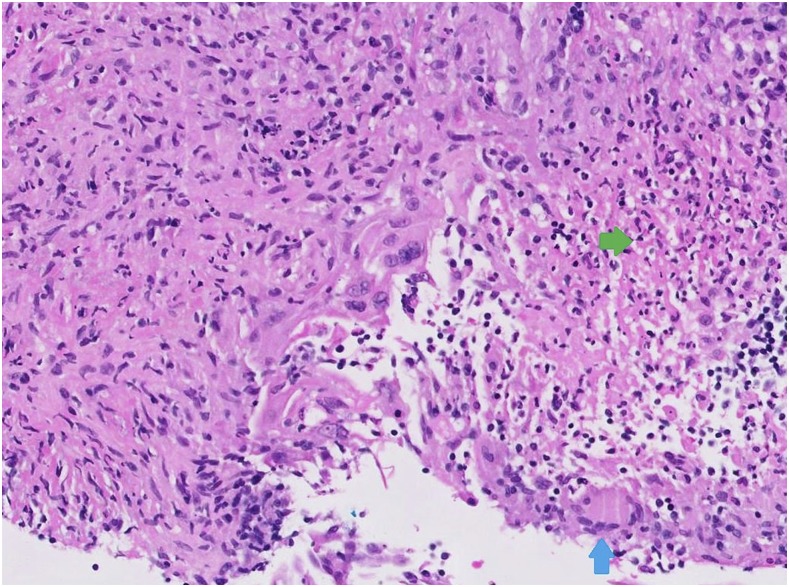
Histopathology reflecting necrotizing granulomatous inflammation (green arrow shows zone of necrosis; blue arrow shows focal multinucleate giant cell). This figure appears in color at www.ajtmh.org.

**Figure 3.**
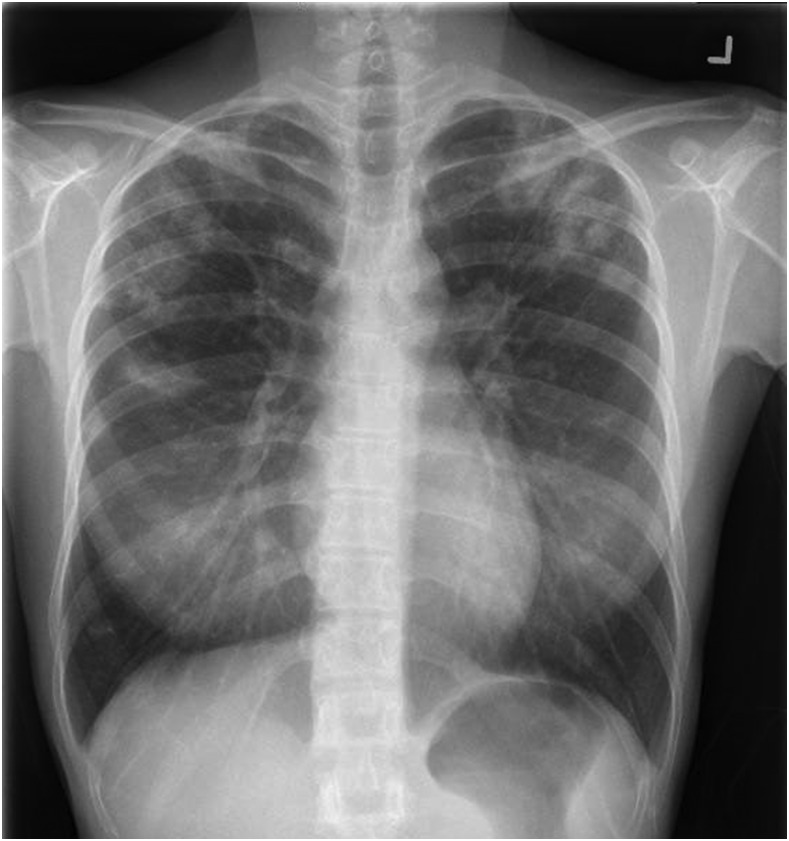
Chest radiograph shows nodular opacities over the upper and mid-zones with cavitation in the left upper zone.

A biopsy was performed with histology, as shown in Figure 2. This revealed necrotizing granulomatous inflammation and focal multinucleated giant cells. Further staining with Ziehl–Neelsen and Grocott’s methenamine silver stains did not show any acid-fast bacilli or fungi (Figure 2). The nucleic acid amplification test (strand displacement amplification) for the *Mycobacterium tuberculosis* complex was positive. A chest radiograph showed multiple nodular opacities over the upper and mid-zones with cavitation in the left upper zone (Figure 3). Sputum for acid-fast bacilli smear was negative, but culture returned positive for the *M. tuberculosis* complex, susceptible to streptomycin, rifampicin, isoniazid, and ethambutol, four weeks later. Her household contacts were called for contact tracing.

Among cases of tuberculosis of the head and neck region, less than 1% is nasopharyngeal in origin.^1^ Large Asian case series reveal a predominance of female patients, with mean age between 30 and 40 years. An association with pulmonary tuberculosis can be found between 8.3% and 55.6% of the cases.^2^ It is thought that patients can develop primary nasopharyngeal tuberculosis, where infection occurs via direct nasal inhalation. In secondary cases, spread occurs directly through the airways from the lungs or via hematogenous or lymphatic routes.^3^ Between 20% and 40% of patients manifest with nasopharyngeal masses,^2^ as with our patient. Although pulmonary and laryngeal diseases have been frequently cited as risk factors for transmissibility of tuberculosis,^4^ the risk of transmission in nasopharyngeal diseases is not well described.
